# Tumorigenic Potential of Olfactory Bulb-Derived Human Adult Neural Stem Cells Associates with Activation of TERT and NOTCH1

**DOI:** 10.1371/journal.pone.0004434

**Published:** 2009-02-11

**Authors:** Patrizia Casalbore, Manuela Budoni, Lucia Ricci-Vitiani, Carlo Cenciarelli, Giovanna Petrucci, Luisa Milazzo, Nicola Montano, Elisabetta Tabolacci, Giulio Maira, Luigi M. Larocca, Roberto Pallini

**Affiliations:** 1 Institute of Neurobiology and Molecular Medicine, CNR, Rome, Italy; 2 Department of Hematology, Oncology and Molecular Medicine, Istituto Superiore di Sanità, Rome, Italy; 3 Institute of Pathology, Catholic University School of Medicine, Rome, Italy; 4 Institute of Neurosurgery, Catholic University School of Medicine, Rome, Italy; 5 Institute of Genetics, Catholic University School of Medicine, Rome, Italy; City of Hope Medical Center and Beckman Research Institute, United States of America

## Abstract

**Background:**

Multipotent neural stem cells (NSCs) have been isolated from neurogenic regions of the adult brain. Reportedly, these cells can be expanded *in vitro* under prolonged mitogen stimulation without propensity to transform. However, the constitutive activation of the cellular machinery required to bypass apoptosis and senescence places these cells at risk for malignant transformation.

**Methodology/Principal Findings:**

Using serum-free medium supplemented with epidermal growth factor (EGF) and basic fibroblast growth factor (bFGF), we established clonally derived NS/progenitor cell (NS/PC) cultures from the olfactory bulb (OB) of five adult patients. The NS/PC cultures obtained from one OB specimen lost growth factor dependence and neuronal differentiation at early passage. These cells developed glioblastoma tumors upon xenografting in immunosuppressed mice. The remaining NS/PC cultures were propagated either as floating neurospheres or as adherent monolayers with mainteinance of growth factor dependence and multipotentiality at late passage. These cells were engrafted onto the CNS of immunosuppressed rodents. Overall, the grafted NS/PCs homed in the host parenchyma showing ramified morphology and neuronal marker expression. However, a group of animals transplanted with NS/PCs obtained from an adherent culture developed fast growing tumors histologically resembling neuroesthesioblastoma. Cytogenetic and molecular analyses showed that the NS/PC undergo chromosomal changes with repeated *in vitro* passages under mitogen stimulation, and that up-regulation of hTERT and NOTCH1 associates with *in vivo* tumorigenicity.

**Conclusions/Significance:**

Using culturing techniques described in current literature, NS/PCs arise from the OB of adult patients which *in vivo* either integrate in the CNS parenchyma showing neuron-like features or initiate tumor formation. Extensive xenografting studies on each human derived NS cell line appear mandatory before any use of these cells in the clinical setting.

## Introduction

Due to their ability to self-renew and to differentiate towards the neuronal phenoype, human adult neural stem cells (NSCs) provide an attractive tool for transplantation-based therapy of neurodegenerative diseases that avoids the ethical issues raised by the use of human embryos. However, proliferation and self-renewal properties make NSCs sensitive targets for malignant transformation [1]. Some evidence suggests that adult mouse NSCs are quite resistant to transform even in high-passage cultures under mitogen stimulation [2]. In contrast, neural precursors from the adult rat subventricular zone (SVZ) have recently been shown to transform into tumorigenic cell lines after expansion *in vitro* [3]. Moreover, several arguments advise caution before grafting NSCs in patients that include, *a*) evidence that glioblastoma may arise *de novo* from the oncogenic transformation of NSCs [1], [4], *b*) common molecular determinants regulating neurogenesis and tumorigenesis [5]–[7], and *c*) generation of glioma-like lesions following growth factor stimulation of the adult SVZ [8].

The forebrain SVZ and the dentate girus of the hippocampus are two areas of persistent neurogenesis in the adult brain. These regions contain dividing cell populations that have been recognized as NSCs and transit amplifying progenitors (TAPs). The former are relatively quiescent cells with the capacity of self-renewal. TAPs proliferate more rapidly and differentiate into migratory neuroblasts and oligodendrocyte precursors. In rodents, TAPs move along the rostral migratory stream to the olfactory bulb (OB). In humans, a lateral ventricular extension of the migratory stream to the OB has recently been demonstrated and NS/progenitor cells (NS/PCs) have successfully been isolated from the OB, which therefore represents an accessible source of neural precursors [9]–[11]. Using xenograft models, we found that human adult OB-derived NS/PCs are capable of initiating tumor formation. Although an oncogenic potential has previously been described in rodent NSCs [3], [12]–[13] and in human adult mesenchymal stem cells [14], the present work provides the first demonstration that human adult NS/PCs arising from normal brain may be tumorigenic *in vivo*.

## Results and Discussion

### Tumorigenic human adult NS/PCs arising from an OB adjacent to meningioma

**Figure 1:**
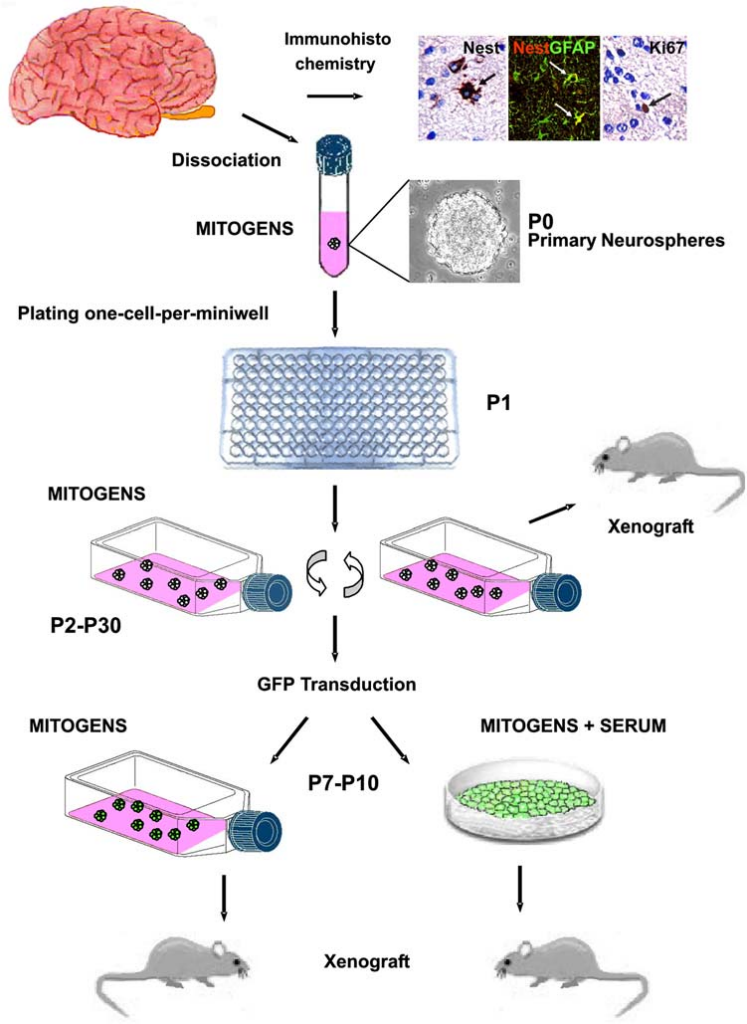
Summary of experimental design. The OB was obtained from adult patients who underwent neurosurgical operations. On immunohistochemical analysis, the human OB was found to contain about 700 to 1000 cells expressing the NS markers nestin and CD133. The nestin-expressing cells colocalize glial fibrillary acid protein (GFAP). These cells are located either within the inner plexiform layer close to the lateral olfactory tract where they show an astrocyte-like morphology, or in the external plexiform layer where they mainly appear as small rounded or unipolar cells. In the external plexiform layer, a few proliferating cells (*n*, 200–300) are detected by Ki67 labeling. Dissociated OB specimens were cultured in serum-free medium supplemented with the mitogens EGF and bFGF. Primary neurospheres were dissociated into single cells and plated one cell per mini-well. Clonal cell cultures were established by dissociation of secondary neurospheres. Clonal cultures from each OB were passaged up to P30 in mitogens. NS/PC cultures which lost growth factor dependence and multipotentiality were assessed for tumorigenicity *in vivo*. At P6, the NS/PCs that maintained growth factor dependence and multipotentiality were transduced to express GFP. The GFP-positive NS/PCs were expanded either as neurospheres in serum-free medium supplemented with mitogens or as adherent monolayers in medium containing mitogens and serum and then engrafted onto the striatum or spinal cord of immunocompromised rodents.

**Figure 2:**
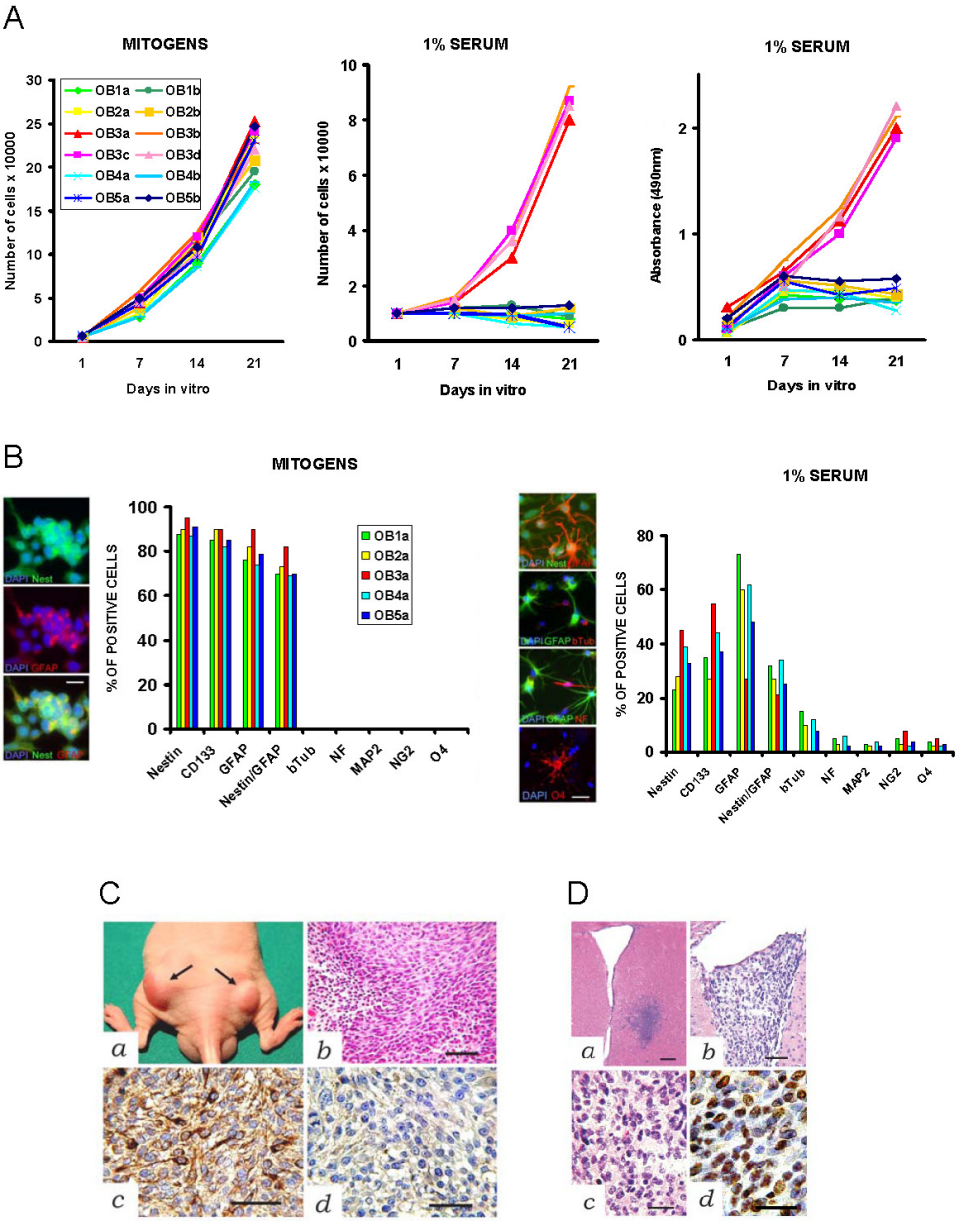
Characterization and grafting of OB-derived NS/PCs cultured as neurospheres. A, Growth curves of NS/PCs (P6) cultured in serum-free medium supplemented with mitogens (*left*) and in medium containing 1% serum without mitogens (*center*). The OB3 cells continue to proliferate in spite of mitogen removal and serum stimulation. Absorbance test for NS/PC viability (*right*). *B*, Immunophenotyping of OB-derived NS/PCs (P6) cultured in serum-free medium supplemented with mitogens (*left*) and in medium containing 1% serum without mitogens (*right*). Double-positive cells for nestin and GFAP were counted positive for each antigen and also for both antigens (nestin/GFAP). The OB3a cells do not differentiate towards the neuronal lineage in response to serum stimulation. *C*, Subcutaneous xenografs of OB3a cells in nude athymic mice. *a*, Subcutaneous nodules two weeks after grafting (arrows in *a*). Histological features of glioblastoma (*b*, H&E). Expression of astrocytic cell marker GFAP (*c*) and negative staining for the neuronal cell marker neurofilament (*d*). *D*, Intracerebral tumor xenografts of OB3a cells in SCID mice. Pattern of brain invasion by OB3a cells one week after grafting into the striatum (*a*, H&E). Low (*b*) and high (*c* and *d*) magnifications of intraventricular anaplastic astrocytoma-like tumor by two weeks after grafting (*b* and *c*, H&E *d*, anti-HNA immunoreaction). *A*, Scale bar 250 µm; *b*, Scale bar 80 µm; *c*, Scale bar 50 µm; *d*, Scale bar 30 µm.

The OB was harvested from five adult patients who had undergone surgery for extracerebral benign lesions (**Table S1**). Unilateral division of the OB, which is often necessary for surgical exposure, is well tolerated by the patients because the olfactory function is preserved. Immunohistochemistry showed that the human adult OB contains a few hundreds of putative NS/PCs (**Fig. 1**). Dissociated OB specimens were cultured in serum-free medium supplemented with the mitogens EGF and bFGF. Under these conditions, the OB cells generated primary neurospheres with latencies that ranged from 6 to 8 weeks (**Table S2**). An exception was Case OB3 where primary neurosphere formation was observed as early as 3 weeks of culturing. Primary neurospheres were dissociated into single cells and plated one cell per mini-well (**Fig. 1**). Clonal cell cultures were established by dissociation of secondary neurospheres and passaged up to P30 in mitogens. The ability to form spheres after serial passaging, the number and diameter of spheres produced during each passage, and the cloning efficiency were similar among different cultures (**Table S2**). Upon removal of mitogens and serum exposure, the NS/PC cultures obtained from four of the OB specimens arrested their growth and gave rise to adherent cells that expressed neuronal, astrocytic, and oligodendrocytic markers (**Figs. 2A–2B**). In contrast, OB3 NS/PC cultures lost both growth factor dependence and potential to differentiate as neurons between P4 and P6. Notably, the OB3 patient harbored a meningioma adjacent to the OB. Losing growth factor dependence and capacity to differentiate by NSCs may indicate transformation. On soft agar assay, an *in vitro* correlate of transformation, the OB3 NS/PCs developed colonies (**Fig. S2**). Then, we assessed tumorigenicity *in vivo* using hetero- and orthotopic xenografts in immunodeficient mice. Two to 3 weeks after grafting, NS/PCs from all OB3 cultures developed subcutaneous tumors with a 88.6 percent take (**Fig. 2C** **and** **Table 1**). Histologically, these tumors showed glioblastoma features, like perinecrotic pseudo-palisading and vascular proliferation. Tumorigenicity of OB3 NS/PCs was demonstrated both at early (P6) and at late passages (P30). Subcutaneous injection of OB1, OB2, OB4, and OB5 NS/PCs resulted in amorphous tissue grafts with embedded scarce cells showing heterogenous morphology and occasional GFAP staining without neoplastic features (not shown). Intracerebral injection of OB3 NS/PCs also produced tumors which developed at 63.1 percent of injection sites by 4 to 6 weeks after grafting (**Fig. 2D** **and** **Table 1**). Histologically, these tumors featured anaplastic astrocytoma with predilection for growing into the ventricles. Intracerebral injection of OB1, OB2, OB4, and OB5-derived NS/PCs did not result in tumor formation (**Table 1**).

**Table 1:** Tumor Formation after Grafting of Human Adult OB-derived NS/PCs in Immunosuppressed Rodents.

Grafted Cells	Xenografts (Tumors/Injection points)		
	Subcutaneous	Intracerebral	Intramedullary
OB1a P6	0/4		
OB1a P8 GFP+		0/3	
OB1a P9	0/2		
OB1b P9	0/2		
OB1b P9 GFP+	0/4	0/3	
OB1a P10	0/2		
OB1a P10 GFP+		0/3	
OB1b P10 GFP+		0/4	
OB1a P18	0/4		
SS-OB1a P7	0/4		
SS-OB1a P9 GFP+	0/2	0/2	0/3
SS-OB1b P9 GFP+		0/2	0/4
OB2a P7	0/4		
OB2a P7 GFP+		0/3	
OB2b P7 GFP+		0/2	
OB2b P9	0/4		
OB2a P8 GFP+		0/4	0/4
OB2b P8 GFP+		0/2	0/5
OB2a P12	0/4		
OB2a P10 GFP+		0/4	
OB2b P18	0/4		
OB2b P10 GFP+		0/2	
SS-OB2a P7	4/4		
SS-OB2a P8 GFP+		3/5	6/7
SS-OB2b P8	0/4		
SS-OB2a P8 GFP+		4/4	
SS-OB2a P10	3/4		
SS-OB2a P10 GFP+	4/4	3/4	12/14
SS-OB2b P10 GFP+	0/4		
OB3a P6	4/4	2/3	
OB3b P6	9/10	3/4	
OB3a P12	4/4		
OB3b P12	2/2		
OB3c P12	4/5	3/4	
OB3b P18	3/4	2/4	
OB3d P18	3/4		
OB3a P30	2/2	2/4	
OB4a P6	0/4		
OB4a P7 GFP+		0/3	
OB4b P6	0/4		
OB4a P12	0/4		
OB4b P12	0/4		
OB4a P10 GFP+		0/4	0/2
SS-OB4a P8	0/4		
SS-OB4b P8 GFP+		0/4	0/3
SS-OB4a P12	0/4		
OB5a P6	0/6		
OB5b P6	0/4		
OB5b P9	0/4		
OB5a P8 GFP+		0/4	
OB5b P8 GFP+		0/3	
OB5a P18	0/4		
SS-OB5a P8	0/4		
SS-OB5a P10 GFP+		0/4	

NS/PCs, neural stem/progenitor cells; OB, olfactory bulb; P, passage in vitro; GFP, green fluorescent protein; SS, serum stimulated.

In principle, taking NS/PCs from patients with pre-existing tumors nearby the organ where the cells are obtained is inappropriate. For example, human adult non-tumorigenic NSCs surrounding low-grade glioma tissue transform *in vitro* into highly tumorigenic cancer stem cells [15]. In patient OB3, errant meningioma cells infiltrating the OB or adhering to its surface might have overwhelmed the NS/PCs in culture. This hypothesis, however, seems unlikely because of the following, *1*) the phenotype of meningioma cells (EMA+/GFAP−/NG2−/O4−) differed both from that of OB3 NS/PCs (EMA−/GFAP+/NG2+/O4+) and from OB3-derived tumor xenografts (EMA−/GFAP+/NG2−/O4−); *2*) sphere generation in serum-free cultures occurs in glioblastoma, anaplastic astrocytoma, medulloblastoma, and ependymoma but not in meningioma, and *3*) meningioma-derived NS/PCs are expected to develop xenografts with the histological appearance of meningioma or sarcoma not of glioblastoma. In brain pathology, concurrent adjacent meningioma and astrocytic tumors have been described raising the hypothesis that meningioma-released agents may work as growth factors for the glial cells of surrounding brain tissue [16]. Thus, the NS/PCs resident in the OB adjacent to meningioma may undergo chronic pressure for growth becoming highly sensitive to mitogens *in vitro*.

### Transformation of human adult NS/PCs following propagation in mitogens and serum

**Figure 3:**
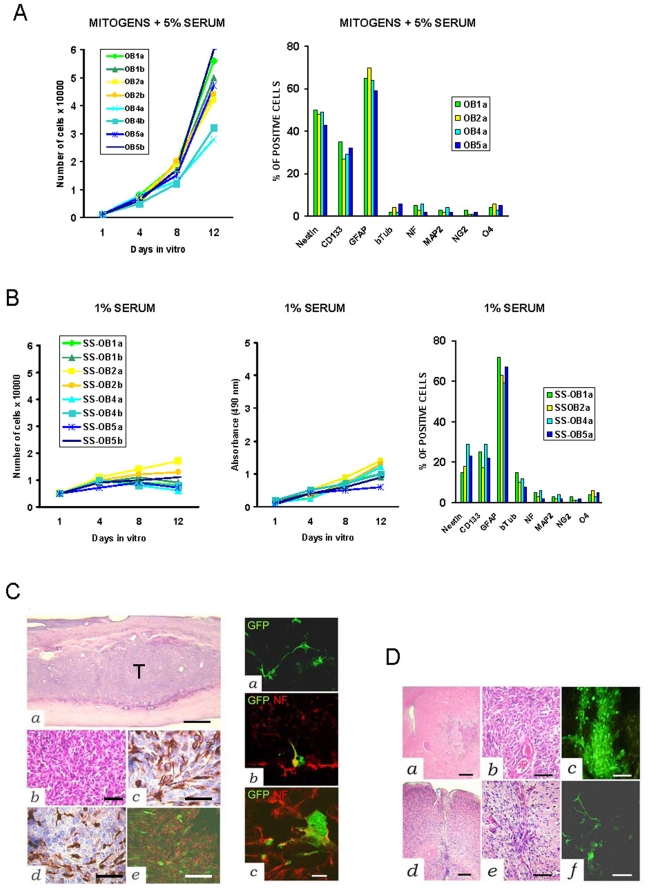
Characterization and grafting of serum-stimulated NS/PCs grown as adherent cultures. *A*, Growth curves (*left*) and immunophenotype (*right*) of NS/PCs (P6) cultured in medium containing mitogens and 5% serum (serum-stimulated, SS-NS/PCs). *B*, Growth curves (*left*), absorbance test (*right*), and immunophenotype (*right*) of SS-NS/PCs cultured in 1% serum. *C*, Grafting of SS-NS/PCs onto the spinal cord of ciclosporine treated rats. Intramedullary tumor developed four weeks after grafting of SS-OB2a cells (*left*). Low (*a*) and high (*b*) magnification sections show a neuroesthesioblastoma-like tumor (T) (H&E) expressing both neurofilament (*c*) and GFAP (*d*). Tumor cells labeled with GFP (*e*; green) stain with the oligodendrocyte cell marker NG2 (red). Homing and differentiation of SS-NS/PCs after grafting onto the spinal cord (*left*). GFP-labeled SS-OB1a cells showing neuronal cytology (*a*). GFP-labeled SS-OB4a cells expressing the neuronal marker neurofilament (GFP green, neurofilament red, merged signal yellow) (*b* and *c*). *D*, Intracerebral grafts of SS-NS/PCs in SCID mice. Brain tumor developed by two weeks after grafting of GFP-labeled SS-OB2a cells (*a–c*; *a–b*, H&E *c*, fluorescence microscopy). Grafted SS-OB1a cells do not form tumor by eight weeks after implantation (*d–f*; *d–e*, H&E *f*, fluorescence microscopy). *Left*. *a*, Scale bar 300 µm; *b–e*, Scale bar 50 µm. *Center*. *a–c*, Scale bar 30 µm. *Right*. *a* and *d*, Scale bar 250 µm; *b–c* and *e–f*, Scale bar 70 µm.

Transplantation technologies of adult human NS/PCs imply strategies where minimal donor material is highly expanded *in vitro* to the adequate cell number before implantation. In general, NSCs can be expanded either as floating neurospheres in serum-free medium supplemented with mitogens or as adherent monolayers in medium containing both mitogens and serum [17]. Neuronal and oligodendroglial differentiation of adherently growing NSCs can be enhanced by growth factor withdrawal and exposure to triiodothyronine (T3) and ascorbic acid [18]. Then, we propagated GFP-positive OB1, OB2, OB4, and OB5 NS/PCs between P7 and P10 either under mitogens or under mitogens and 5% serum (**Fig. 1**). In mitogens and serum, the NS/PCs became adherent, continued to proliferate, and either maintained an undifferentiated phenotype or differentiated, mainly as astrocytes (**Fig. 3A**). When such adherent serum-stimulated (SS) NS/PCs were returned to serum-free medium with mitogens, they formed floating neurospheres within one week maintaining their clonal efficiency. Upon removal of mitogens and exposure to 1% serum supplemented with T3 and retinoic acid, the SS-NS/PCs slowered down their growth and further differentiated towards the neuronal, astrocytic, and oligodendrocytic lineages (**Fig. 3B**). Aberrant coexpression of neuronal and glial markers by the SS-NS/PCs was not seen.

To examine the behavior of NS/PCs in the CNS environment, we engrafted GFP-positive NS/PCs, which had been expanded either as neurospheres or as adherent monolayers, onto the spinal cord of ciclosporine treated rats or onto the striatum of SCID mice. Surprisingly, 85.7 percent of the rats engrafted onto the spinal cord with the clonal SS-OB2a NS/PCs showed progressive palsy of their hindlimbs by 2 to 4 weeks after grafting. These animals developed highly infiltrating intramedullary tumors that histologically were reminiscent of neuroesthesioblastoma, a malignant neoplasm of the OB that is supposed to arise from an ancestral neuroblast (**Fig. 3C** **and** **Table 1**). The tumor xenografts expressed markers for neuronal, astrocyte, and oligodendrocyte cells. Intracerebral grafting of the SS-OB2a NS/PCs also resulted in tumor formation with 76.9 percent take (**Fig. 3D** **and** **Table 1**). Importantly, the clonally-derived OB2a NS/PCs which had been expanded as neurospheres homed in the host parenchyma showing ramified morphology and neuronal marker expression without generating any tumor (**Figs. 3C–3D**). Similar findings were seen in animals engrafted with OB1, OB4, and OB5 NS/PCs irrespective the technique used for their propagation *in vitro*. Thus, the oncogenic transformation of human adult NSCs may occur whether a combination of expansion/selection stimuli, like mitogens and serum, are simultaneously applied to these cells *in vitro*. Consistently, mouse embryonic NS cells that have simultaneously been serum-differentiated and expanded with FGF, develop tumors *in vivo* [1].

### Cytological and molecular characteristics of tumorigenic NS/PCs

**Figure 4:**
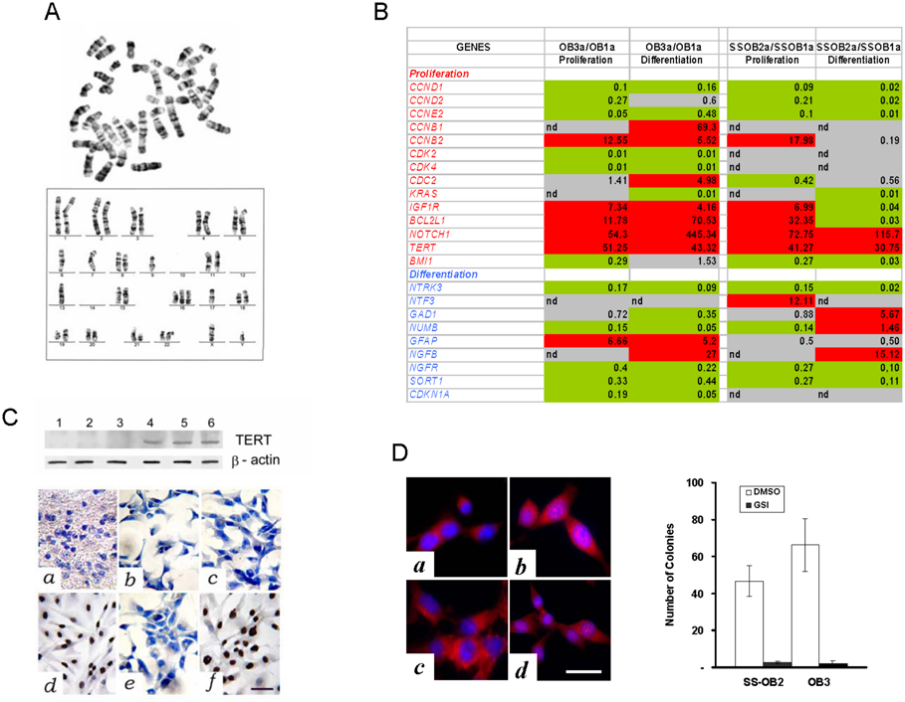
Cellular features and molecular profiling of tumorigenic OB-derived NS/PCs. *A*, Metaphase spread and manually aligned karyotype of OB3a NS/PCs (P6) showing remarkable chromosomal rearrangements. *B*, Expression array analysis performed with RNA extracted from NS/PCs (P7–P8). Gene expression in tumorigenic OB3a and SS-OB2a cells is presented relative to the non tumorigenic OB1a and SS-OB1a cells, respectively, both under proliferating culture conditions and under serum-induced differentiation. Upregulated genes (2 folds, red), downregulated genes (2 folds, green), unchanged genes (gray). *C*, Expression of hTERT protein in OB-derived NS/PCs. Western blot analysis of HUVEC (lane 1), OB1a (lane 2), OB2a (lane 3), SS-OB2a (lane 4), OB3a (lane 5), and TB10 human glioblastoma (lane 6) cells. Immunohistochemical analysis of hTERT expression in the human adult OB and OB-derived NS/PCs. The hTERT protein is absent in the adult OB (*a*) as well as in the non-tumorigenic OB1a (*b*), OB2a (*c*), and SS-OB1a (*e*) cells. hTERT is strongly expressed in the nuclei of both tumorigenic OB3a (*d*) and SS-OB2a (*f*) NS/PCs. Scale bar 40 µm. *D*, Immunofluorescence analysis of NOTCH1 expression (*left*) in OB1a (*a*), OB3a (*b*), OB2a (*c*), and SS-OB2a (*d*) NS/PCs. NOTCH1 signaling is required for the formation of colonies in soft agar (*right*). Exposure to γ-secretase inhibior X (GSI) after seeding in soft agar significantly reduced clonigenic potential of tumorigenic OB3a and SS-OB2a NS/PCs (*P*<0.0001, Student *t*-test).

Somatic stem cells are thought to possess proficient mechanisms that allow replicative potential and chromosomal stability. However, chromosomal rearrangements have been detected in long-term expanded adult murine NSCs which apparently do not result in a malignant phenotype [2]. We performed kariotype analysis on NS/PCs at regular time points and found chromosomal rearrangements with repeated passages under mitogen stimulation (**Figs. 4A** **and S1**). Chromosomal changes were found both in tumorigenic and in non-tumorigenic NS/PCs suggesting that these cells need additional requirements to achieve tumorigenicity *in vivo*. It has been reported that the tumor-like growth properties of the stem cells associate with changes either in oncosuppressors or in oncogenes [2], [5]–[6], [15]. Then, we set up a custom real-time RT-PCR array to analyze the expression of 92 mRNAs related with cell proliferation and cancer in tumorigenic relative to non tumorigenic NS/PCs both under proliferating culture conditions and under serum-induced differentiation (**Fig. 4B**). Relative to the non tumorigenic OB1a and SS-OB1a NS/PCs, tumorigenic OB3a and SS-OB2a NS/PCs showed upregulation of genes related to cell proliferation and inhibiting apoptosis, though solely hTERT and NOTCH1 were overexpressed independently from mitogen stimulation. Tumorigenic OB3a and SS-OB2a NS/PCs did express the hTERT protein, which was undetectable in non tumorigenic NS/PCs, consistent with that reported in normal NS cells (Fig. 4C) [**19**]. Immunofluorescence with anti-NOTCH1 antibody on tumorigenic OB3a and SS-OB2a NS/PCs demonstrated either increased cytoplasmic staining or abnormal nuclear staining (**Fig. 4D**). Following NOTCH1 blockade with the γ-secretase inhibitor X (GSI), OB3a and SS-OB2a NS/PCs lost their ability to form soft-agar colonies suggesting a functional role of NOTCH1 in tumorigenicity of these cells (**Figs. 4C** **and S2**). Although the xenografts grown after injection of OB3a and SS-OB2a NS/PCs were histologically reminiscent of different tumors, in both of them molecular analyses pointed to hTERT and NOTCH1 as critical pathways. Telomerase is highly expressed in the majority of human cancers including glioblastoma, where it is believed to contribute to tumor progression because telomerase-dependent telomere maintenance provides cells with an extended proliferative potential [20]. Glioblastoma stem cells, which express telomerase under proliferating serum-free conditions, transiently lose telomerase activity in serum-containing media; however, these cells regain telomerase at passages coincident with their exponential growth phase [21]. NOTCH is known to promote the proliferation of nonneoplastic NSCs and to inhibit their differentiation; it is also highly activated in embryonal brain tumors, such as medulloblastoma, where it is required both for maintaining the stem cell fraction *in vitro* and for tumor formation *in vivo* [**22**]. Up-regulation of hTERT and NOTCH1 in both tumorigenic OB3a and SS-OB2a NS/PCs suggests that a common mechanism may underly the malignant transformation of these cells, and that the histological differences between the OB3a-derived glioblastoma and the SS-OB2a-derived neuroesthesioblastoma may reflect different stages at which the NS/PCs have undergone neoplastic transformation in culture. In the OB3a-derived glioblastoma, the tumorigenic hit may have occurred in an astrocytic-committed precursor cell, whilst in the SS-OB2a-derived neuroesthesioblastoma the cell of origin may be a less differentiated NS/PC that has retained its multipotentiality.

Gene therapy trials using human hematopoietic stem cells after retroviral transduction have demonstrated a risk of insertional mutagenesis and oncogenic transformation [23]. However, we do not believe that that the tumorigenic transformation of the OB-derived NS/PCs may be a consequence of the use of the lentiviral vector that integrated the GFP gene into the genome of these cells. The following arguments do not favour this hypothesis, *1*) the OB3 cells, which *in vivo* gave origin to glioblastoma-like tumors, were not transduced with lentivirus to express GFP; *2*) both the OB2 cells and the SS-OB2 cells were transduced with lentivirus, however, only the latter cells developed tumor *in vivo*, whilst the GFP-positive OB2 cells did not; and *3*) the lentivirally transduced OB1, OB4, and OB5 NS/PCs were not tumorigenic *in vivo*.

To conclude, human adult NS/PCs cultured under mitogen stimulation are prone to develop chromosomal rearrangements. *In vivo* tumorigenicity is heralded by, *1*) short latency in primary neurosphere formation, *2*) persistent growth after removal of mitogens, *3*) loss of serum-induced neuronal differentiation, and *4*) up-regulation of hTERT and NOTCH1. The tumorigenic transformation of human adult NS/PCs isolated from an OB adjacent to meningioma raises the possibility that unusual levels of growth factors in the *in situ* condition, i.e. prior to *ex vivo* culture, may prime tumorigenicity. This indicates that the tumorigenic potential of the OB3 NS/PCs may be a specific feature of this cell line and not generalizable. In the case of SS-OB2 NS/PCs, however, specific culture conditions seem critical to transformation. Mitogens used simultaneously with factors favouring cell specification may disrupt the regulatory mechanisms that control self-renewal of NSCs and differentiation of TAPs. Therefore, culturing techniques where both proliferation and differentiation of NS/PCs are simultaneously enhanced should be evaluated further in future and discouraged if confirmed as linked to *in vivo* tumorigenicity.

## Materials and Methods

### Isolation, Culturing, and Immunophenotyping of NS/PCs

The OB was harvested from adult patients undergoing craniotomy at the Institute of Neurosurgery, Catholic University, Rome (**Table S1**). Informed consent was obtained according to protocols approved by the Ethical Commettee of the Catholic University. Immediately after removal, the OBs were dissociated in Papain 0,1% (Sigma-Aldrich, St. Louis, MO) for 30 minutes at 37°C. Dissociated cells were cultured in the presence of human recombinant EGF (20 ng/ml; PeproTech, Rocky Hill, NJ), human recombinant bFGF (10 ng/ml; PeproTech), and LIF (20 ng/ml; Immunological Sciences, Rome, Italy) in DMEM/F12 (1∶1) serum-free medium (Invitrogen, Carlsband, CA) containing L-glutamine 2 mM, glucose 0.6%, putrescine 9.6 ug/ml, progesterone 0.025 mg/ml, sodium selenite 5.2 ng/ml, insulin 0.025 mg/ml, apo-transferrin sodium salt 0.1 mg/ml, sodium bicarbonate 3 mM, Hepes 5 mM, BSA 4 mg/ml, heparin 4 ug/ml [24]. Primary neurospheres were dissociated with Accutase (Invitrogen) for 4 minutes at 37°C, serially diluted, and plated one cell per mini-well onto 96-well plates. Mini-wells containg one single cell were marked after microscopic confirmation and assessed for secondary neurosphere generation after one week. Secondary neurospheres were subsequently dissociated, plated at the density of 10^3^ cells/cm^2^ in serum-free medium containing EGF and bFGF, and passaged up to P30. All experiments were done on at least two clonal cultures from each OB. Between P7 and P10, parallel cultures were established in which cells were grown as adherent monolayers in medium containing EGF and bFGF supplemented with 5% fetal calf serum (Hyclone, Logan, UT). For cell growth experiments, dissociated cells were plated on Matrigel at the density of 10^3^ cells/cm^2^ either in serum-free medium containing EGF and bFGF or in medium where mitogens were replaced with 1% serum or in medium containing mitogens and 5% serum (Hyclone). Cells were counted with hemacytometer every 48 hours. Cell viability was determined colorimetrically by MTS-assay (Supplementary Methods S1). Differentiation assays were performed by 14 days after plating on Matrigel coated glass coverslips in the absence of EGF and bFGF and in the presence of 1% fetal calf serum (Hyclone) supplemented with 3′-5′-cyclic adenosine monophosphate (cAMP) 50 µM, all- *trans* retinoic acid 5 µM (Sigma Aldrich), and triiodothyronine (T3) 30 nM (Sigma Aldrich). Immunostaining of NS/PCs was performed as described [15]. We used antibodies against nestin (Chemicon, Temecula, CA), CD133 (CD133/2; Miltenyi, Bergisch, Germany), GFAP (Dako, Glostrup, Denmark), β tubulin III (Chemicon), neurofilament RT-97 (Developmental Studies Hybridoma Bank, Iowa City, IA), MAP2 (Chemicon), NG2 (Chemicon), O4 (Chemicon), hTERT (Novocastra Laboratories), and NOTCH1 (Chemicon).

### Generation of Fluorescent NS/PCs

Enhanced green fluorescent protein (GFP) gene transfer in the NS/PCs was performed at P6 using a variant of third generation lentiviral vectors as described [25].

### Grafting of NS/PCs in Immunodeficient Rodents

Studies involving animals were approved by the Ethical Committee of the Catholic University School of Medicine, Rome. The NS/PCs were grafted either subcutaneously in nude athymic mice, or into the brain of severe combined immunodeficient (SCID) mice, or onto the spinal cord of ciclosporine treated rats (Supplementary Methods S1). For implantation, the NS/PC cultures were splitted 24–48 hours prior to transplant and injected as single cell suspensions. After two to 6-week survival, the animals were sacrificed with an overdose of barbiturate. Either the subcutaneous graft or brain or spinal cord was removed and processed for histology as described [25].

### Immunohistochemistry

Immunohistochemistry was performed on deparaffinized sections using the avidin-biotin-peroxidase complex methods as described [25]. The following primary antibodies were used, anti-GFAP (Ylem, Avezzano, Italy), anti-neurofilament (Ylem), anti-NG2 (Chemicon), anti-CD133/1 (Miltenyi), anti-nestin (Santa Cruz Biotecnology), anti-human nuclei antigen, (HNA; Chemicon), anti-epithelial membrane antigen (EMA; Ylem), anti-Ki67 (Dakocytomation), anti-hTERT (Novocastra Laboratories). Endogenous biotin was saturated by biotin blocking kit (Vector). For antigen retrieval, paraffin sections were microwave-treated in 0.01 M citric acid buffer at pH 6.0 for 10 min. For hTERT antigen retrieval, paraffin section were microwave-treated in EDTA buffer at pH 8.0 for 10 min.

### Chromosome analysis

NS/PC cultures at P3–P8 were incubated in medium containing 10 ng/ml colcemid for 18 hours. The cultures were then lifted and centrifuged. Pellets were osmotically shocked with 0.075 M KCl and fixed with 3∶1 methanol∶glacial acetic acid. Standard cytogenetic G bands were performed and a mean of 20 methaphases per cell lines were analyzed.

### Macroarray Analysis

We used a 7900HT instrument equipped with SDS2.2 software to perform a custom real-time RT-PCR array (Microfluidic Card, Applied Biosystems, CA). Briefly, cells were plated on Matrigel pre-coated 100 mm dishes and processed as described above. Preparation of total RNA and cDNA was performed using Ribo Pure kit (Ambion, Austin, TX) and high capacity cDNA Reverse Transcriptase kit (Applied Biosystems), respectively. For data analysis, the mathematical process for deriving relative quantification values was used as described by the manufacturer's guide (Applied Biosystems).

### Western Blot

Cell pellets were lysated in a modified RIPA buffer (Tris-HCl 10 mM pH 7.5, NaCl 10 mM, NP-40 0.2%, EGTA 1 mM, EDTA 1 mM, DTT 1 mM and protease inhibitor cocktail; Sigma-Aldrich) on ice for 5–10 min. Nuclear extracts were resuspended in Urea buffer (10 M Urea, Tris-HCl 50 mM pH 7.5, DTT 25 mM) sonicated and normalized using Bradford Assay (Promega Corp). Protein extracts were analyzed by polyacrylamide gel electrophoresis and Western blot. Proteins were probed with rabbit polyclonal anti-TERT (1∶1000; Santa Cruz Biotecnology) and monoclonal anti-β-actin (1∶5000; Sigma-Aldrich). As control, HUVEC cells at passages 4 to 5 (Bio-Wittaker, Walkersville, MD) and TB10 glioblastoma cells were used.

## Supporting Information

Table S1 (0.02 MB DOC)

Table S2 (0.02 MB DOC)

Methods S1 Supplementary Materials and Methods
(0.04 MB DOC)

Figure S1 Methaphase spreads and manually aligned karyotypes on NS/PCs. Both the OB2a NS/PCs, which do not develop tumor in vivo, and the SS-OB2a NS/PCs, which are tumorigenic in vivo, show remarkable chromosomal changes at P8–P10, consisting mainly in deletions. Normal karyotype of OB4a NS/PCs at P4.
(9.73 MB TIF)

Figure S2 Soft agar assay (see Supplementary Methods). The OB2a, SS-OB2a, and OB3a NS/PCs were seeded with a mixture of Top Agar (0,5%)-proliferation medium on top of the base layer. The plates were then incubated at 37° in humidified incubator for 3–4 weeks and colonies were counted. Every week fresh medium mixed with Top-agar was added together with 5 µmol/L γ-secretase inhibitor X (GSI; L-685.458) or DMSO as control. Three plates for each NSC/PC culture were used.
(9.58 MB TIF)
