# Supplementary material for: Tumorigenic Potential of Olfactory Bulb-Derived Human Adult Neural Stem Cells Associates with Activation of TERT and NOTCH1
Source: PLoS One. 2009 Feb 11;4(2):e4434. doi: 10.1371/journal.pone.0004434 (PMC2637538; doi:10.1371/journal.pone.0004434)
Supplement: Table S1 — (0.02 MB DOC) [file pone.0004434.s001.doc]

Supplementary Table S1. General Features of Donor Patients

| **Case Code** | **Age/Sex** | **Diagnosis** | **Outcome** |
| --- | --- | --- | --- |
| OB1  OB2  OB3  OB4  OB5  OB6 *  OB7 *  OB8 * | 45/M  39/M  46/F  36/F  48/M  53/F  65/M  33/F | Olfactory groove meningioma (*a*)  Anterior comunicating artery aneurysm  Diaphragma sellae meningioma (*b*)  Anterior comunicating artery aneurysm  Fibrous dysplasia of the orbit  Anterior comunicating artery aneurysm  Olfactory groove meningioma (*c*)  Suprasellar pituitary adenoma (*d*) | NED  NED  NED  NED  AWD  NED  DUD  NED |

*a,*  WHO grade I, phenotype EMA+, VIM+, GFAP-;

*b,* WHO grade I, phenotype EMA+, VIM+, GFAP-;

*c,* WHO grade I, phenotype EMA+, VIM+, GFAP-;

*d,* Phenotype PRL+;

*, Specimens used for immunohistochemical analysis.

NED, no evidence of disease; AWD, alive with disease; DUD, died of unrelated diseases.
