# Supplementary material for: Tumorigenic Potential of Olfactory Bulb-Derived Human Adult Neural Stem Cells Associates with Activation of TERT and NOTCH1
Source: PLoS One. 2009 Feb 11;4(2):e4434. doi: 10.1371/journal.pone.0004434 (PMC2637538; doi:10.1371/journal.pone.0004434)
Supplement: Table S2 — (0.02 MB DOC) [file pone.0004434.s002.doc]

Supplementary Table S2.

| **Case Code** | **Latency (weeks) *** | **Clonal Cultures** | **Number of Spheres** | | **Diameter of Spheres (μm)** | **Clonogenic Index (%)** |
| --- | --- | --- | --- | --- | --- | --- |
|  |  |  | **P6** | **P30** |  |  |
| OB1  OB2  OB3  OB4  OB5 | 8  6  3  7  6 | OB1a  OB1b  OB2a  OB2b  OB3a  OB3b  OB3c  OB3d  OB4a  OB4b  OB5a  OB5b | 1. 250 2. 230 3. 265 4. 260 5. 180 6. 205 7. 220 8. 230 9. 210 10. 250 11. 200   210 190 | | 67.0+ 8.9  75.3+7.6  79.2+ 4.5  68.1+9.0  64.9+13.6  65.4+9.8  59.3+10.1  62.7+4.9  77.5+8.5  66.0+9.2  69.2+3.9  64.1+7.6 | 5.2  5.0  5.5  4.7  4.2  4.1  5.6  4.9  5.1  5.5  4.3  5.0 |

*, Latency for Generation of Primary Spheres.
