# Supplementary material for: Tumorigenic Potential of Olfactory Bulb-Derived Human Adult Neural Stem Cells Associates with Activation of TERT and NOTCH1
Source: PLoS One. 2009 Feb 11;4(2):e4434. doi: 10.1371/journal.pone.0004434 (PMC2637538; doi:10.1371/journal.pone.0004434)
Supplement: Methods S1 — Supplementary Materials and Methods (0.04 MB DOC) [file pone.0004434.s003.doc]

**Supplementary Methods**

*MTS-Assay*

MTS is a tetrazolium compound which is reduced by the cells into a colored formazan product that is soluble in tissue culture medium. The quantity of formazan product is directly proportional to the number of living cells in culture. The cells were plated as described, the MTS solution (Promega Corporation, Madison, WI) was added, and cultures were incubated for 1 hour at 37°C. The absorbance was measured in 490 nm using a spettrophotometer.

*Soft Agar Assay*

The base layer (BactoAgar Difco-BD) was made with 0,8% agarose-proliferation medium and 10^4^ cells were seeded with a mixture of Top Agar (0,5%)-proliferation medium on top of the base layer. The plates were then incubated at 37° in humidified incubator for 3 weeks and colonies were counted. Every week fresh medium mixed with Top-agar was added together with the γ-secretase inhibitor X (GSI; 5mmMol/L; L-685.458, Calbiochem-Germany) and DMSO as control. Three plates for each NS/PC culture were used.

*Subcutaneous, Spinal Cord, and Brain Implantation of NS/PCs in Immunodeficient Rodents*

Nude athymic mice (male, 4-6 weeks of age; HDS-athymic nude mice, Harlan, Udine, Italy), ciclosporine treated rats (male, 150-200 gm; Wistar, Catholic University Breeding Laboratoty, Rome, Italy), and SCID mice (both sexes, 4 weeks, Charles River, Lecco, Italy) were used. For subcutaneous grafting, 4×10^5^ NS/P cells were resuspended in 0.1 ml of cold PBS, the suspension was mixed with an equal volume of cold Matrigel (BD Bioscience, Bedford, MA) and injected in nude mice. For grafting onto the SC, ciclosporine treated rats were anesthetized with intraperitoneal diazepam (2 mg/100 g) followed by intramuscular ketamine (4 mg/100 g). Laminectony was performed at T6-T7 levels and 8×10^4^ GFP^+^ NS/PCs resuspended in 4μl of serum free DMEM were slowly injected using a glass pipette with a sharp beveled tip 100 μm in diameter which was connected to a Hamilton microdrive syringe. For intracranial grafting, the animal skulls were immobilized in a stereotactic head frame, and a burr hole was made 2 mm right of the midline and 1 mm behind the coronal suture. The tip of a 10μl-Hamilton microsyringe was placed at a depth of 3.5 mm from the dura and 2×10^5^ GFP^+^ NS/PCs were injected. After grafting, the animals were kept under pathogen-free conditions in positive-pressure cabinets (Tecniplast Gazzada, Varese, Italy) and observed weekly both for the visual appearance of subcutaneous nodules and for neurological signs.

*Histology of Xenografts*

Specimens were fixed in 4% paraformaldehyde, embedded into paraffin, cut in 5 μm thick sections, and stained with H&E. Animals grafted with fluorescent cells were deeply anesthetized and transcardially perfused with 0.1 M PBS (pH 7.4), followed by 4% paraformaldehyde in 0.1 M PBS. The SC or brain was removed, stored in 30% sucrose buffer overnight at 4°C, and cryotomed at 20 μm. Sections were collected in distilled water, mounted on slides, and cover-slipped with Eukitt. Images were obtained with a Laser Scanning Confocal Microscope (IX81, Olympus Inc, Melville, NY).
