# Supplementary figures and images for: Tumorigenic Potential of Olfactory Bulb-Derived Human Adult Neural Stem Cells Associates with Activation of TERT and NOTCH1
Source: PLoS One. 2009 Feb 11;4(2):e4434. doi: 10.1371/journal.pone.0004434 (PMC2637538; doi:10.1371/journal.pone.0004434)

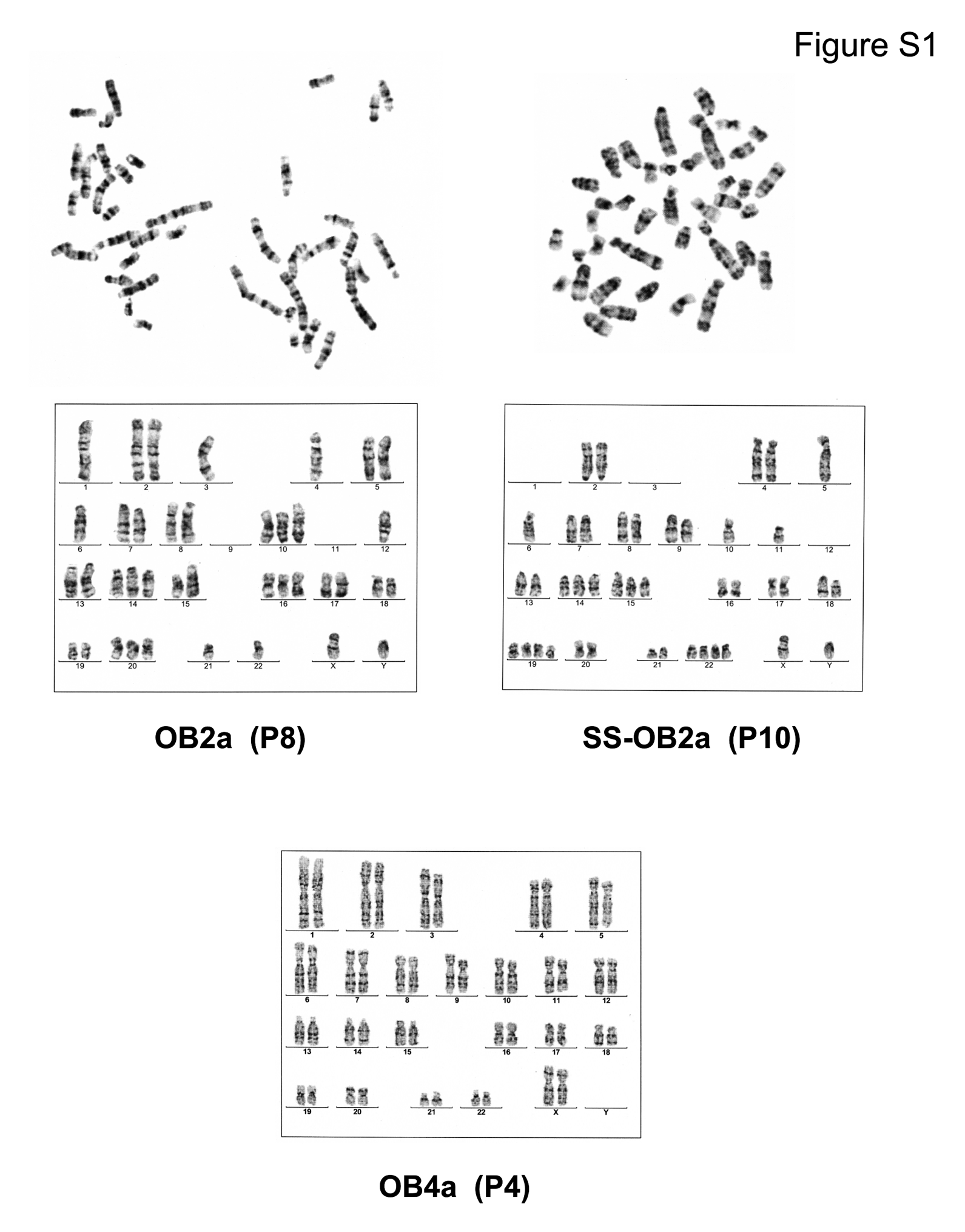

Supplement: Figure S1 — Methaphase spreads and manually aligned karyotypes on NS/PCs. Both the OB2a NS/PCs, which do not develop tumor in vivo, and the SS-OB2a NS/PCs, which are tumorigenic in vivo, show remarkable chromosomal changes at P8–P10, consisting mainly in deletions. Normal karyotype of OB4a NS/PCs at P4. (9.73 MB TIF) [file pone.0004434.s004.tif]

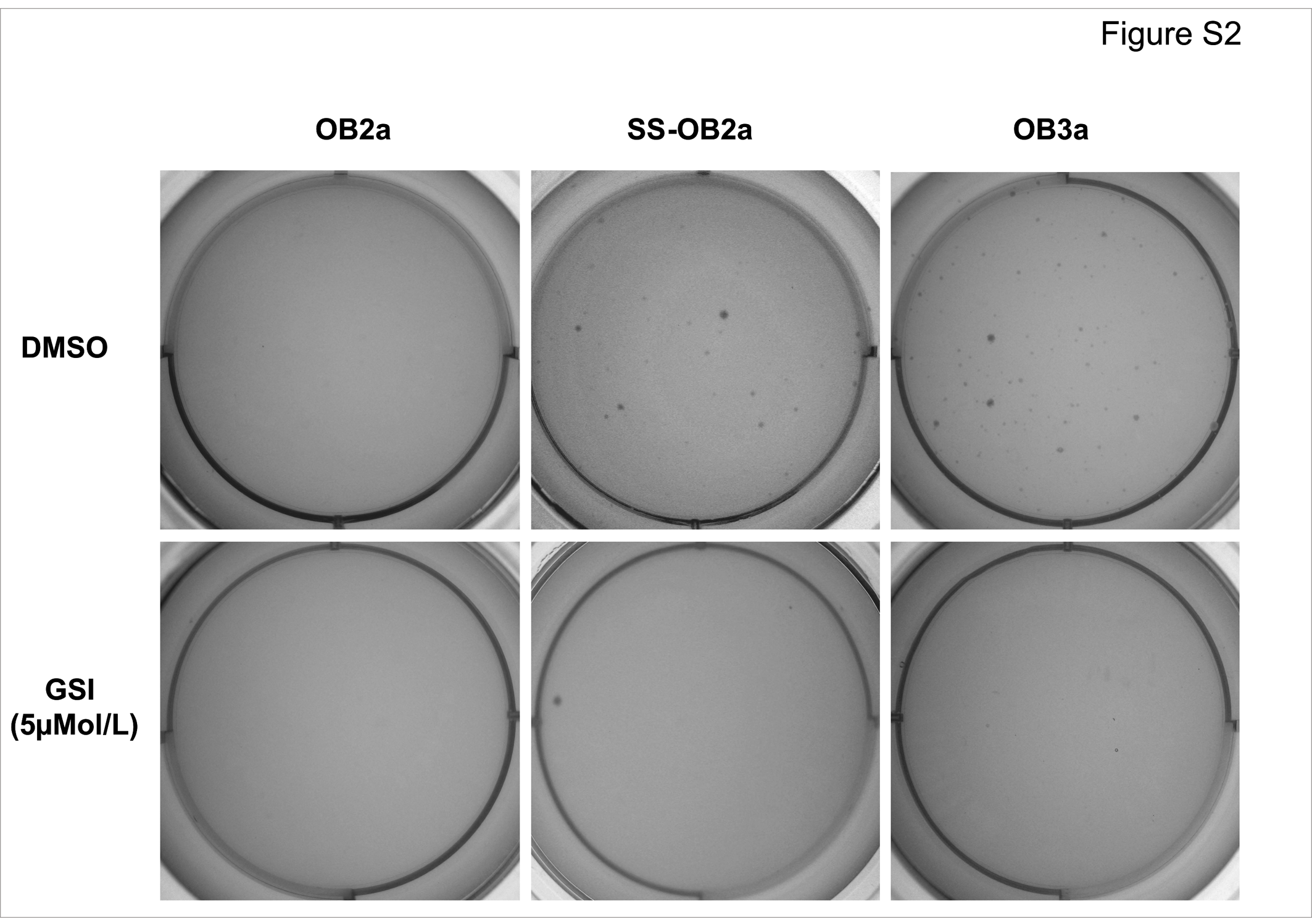

Supplement: Figure S2 — Soft agar assay (see Supplementary Methods). The OB2a, SS-OB2a, and OB3a NS/PCs were seeded with a mixture of Top Agar (0,5%)-proliferation medium on top of the base layer. The plates were then incubated at 37° in humidified incubator for 3–4 weeks and colonies were counted. Every week fresh medium mixed with Top-agar was added together with 5 µmol/L γ-secretase inhibitor X (GSI; L-685.458) or DMSO as control. Three plates for each NSC/PC culture were used. (9.58 MB TIF) [file pone.0004434.s005.tif]
